# Relations of the Number of Functioning Distractors With the Item Difficulty Index and the Item Discrimination Power in the Multiple Choice Questions

**DOI:** 10.7759/cureus.42492

**Published:** 2023-07-26

**Authors:** Girish R Chauhan, Bhoomika R Chauhan, Jayesh V Vaza, Pradip R Chauhan

**Affiliations:** 1 Department of Oral Pathology and Maxillofacial Surgery, Government Dental College, Jamnagar, Jamnagar, IND; 2 Department of Obstetrics and Gynaecology, Banas Medical College and Research Institute, Palanpur, IND; 3 Department of Orthopaedics, Lallubhai Gordhandas Medical College, Ahmedabad, IND; 4 Anatomy, All India Institute of Medical Sciences, Rajkot, Rajkot, IND

**Keywords:** item analysis, difficulty index, discrimination index, distractor effectiveness, functioning distractor

## Abstract

Background

Multiple choice questions (MCQ) are used nowadays in summative assessments for certification of courses, in a competitive examination, and for recruitment. A single-best-answer stem-type MCQ is one of the formats most commonly used for this purpose; it contains a question and options from which the examinee needs to select the correct answer. Item analysis is used for the determination of the quality, validity, and reliability of the MCQ. Item difficulty index, item discrimination power, and distractor effectiveness are critical for the quality of the MCQs.

Aim

This study was conducted to estimate the effect of distractor effectiveness on the item difficulty index and item discriminating power.

Method

A test paper consisting of 200 single-best-answer stem-type MCQs with four options responded to by 400 medical undergraduates was analyzed for item difficulty index, item discriminating power, and distractor effectiveness with established item analysis formulas. The effect of distractor effectiveness on item difficulty index and item discriminating power was statistically analyzed using Epi-Info 7^TM ^software.

Result

The mean item difficulty index, item discriminating power, and distractor effectiveness were 57.75% ± 28.65%, 0.4388 ± 0.36, and 84.17%, respectively (p<0.05). The item difficulty index was higher for items with single-functioning distractors in comparison to items with three-functioning distractors. Item discriminating power was higher in items with three functioning distractors in comparison to items with one and two functioning distractors.

Conclusion

Two or three functioning distractors show an appropriate item difficulty index and item discriminating power. In addition, item discriminating power is lower for the easy MCQs. Two to three distractors are appropriate to construct a quality MCQ.

## Introduction

Multiple choice questions (MCQ) are used in summative assessments for certification of courses, competitive examinations for admission in professional courses like medicine, and recruitment in government services [1-5].

Single-best-answer multiple-choice questions (MCQs) consist of the stem (a question), the distractors (two or more options from which examinees must choose the correct option), and the key (one correct or best response) [6]. Item analysis is the process of collecting, summarizing, and using information from students’ responses to assess the quality of test items [6,7]. Item analysis is the simple process through which an item's reliability and validity can be tested [8].

Classical test theory and item response theory methods are used for item analysis, of which classical test theory is the most commonly practiced. Item difficulty index (P score), item discrimination index (D score), and distractor effectiveness are used in classical test theory to assess the items [7].

The difficulty index (P Score) is also known as the ease index; it ranges from 0-100%; the higher the percentage, the easier the item. The difficulty index indicates the percentage of students who correctly answered the item. The recommended range of difficulty is from 30 to 70%; items having a P score of less than 30% are considered difficult items, and items having a P score of more than 70% are considered easy items [9].

The ability of an item to distinguish between high and low scorers is calculated by the item discrimination index (item discriminating power, D score, DI) [10].

The item discrimination index ranges between -1.00 and +1.00; the discriminating power for the excellent discriminator is ≥0.35 while the poor discriminator has DI ≤0.20; the discriminating power between 0.20 and 0.35 is acceptable. If low-performing students got a specific item correct more than the high scorers, then the item discrimination index will show a negative result (<0.00) [8].

Distractor analysis provides information regarding the individual distractors and key of a test item. The examiner can modify or remove the specific item for subsequent exams using distractor analysis [11]. The quality of distractors determines the examinee’s performance and assesses the precision of knowledge [12].

Non-functioning distractors are options that are selected infrequently (<5%) by examinees or otherwise do not perform as expected. As such, these options should be removed from the item or replaced with a more plausible option [13].

The effect of distractor effectiveness on item difficulty index and item discrimination power can help the paper setter frame multiple-choice questions with a sufficient number of functional distractors with a test-demanding difficulty index and discriminating power.

## Materials and methods

Data collection

This cross-sectional study was performed in December 2020 in Gujarat. This study was exempt from institutional ethical committee approval as it did not involve any intervention with living or dead humans or animals. A test paper with a 0.91 (>0.70) Kuder-Richardson (KR20) reliability index was used, which was constructed confidentially by four experienced medical academicians from different places in Gujarat.

A test paper contained 200 single-best-answer stem-type multiple-choice questions from different medical subjects like medicine, surgery, obstetrics, orthopaedics, pediatrics, radiology, skin, psychiatry, anatomy, physiology, biochemistry, pharmacology, microbiology, pathology, ophthalmology, otolaryngology, and community medicine.

Four hundred medical graduates (327 males and 73 females; mean age 23 ± 0.83) who completed the internship in 2019 participated in the computer-based online examination. The students enrolled on a voluntary basis after receiving informed consent from various medical colleges in Gujarat. The examination was conducted in Ahmedabad as a computer-based test. The examinees were given three hours to complete the examination. The correct response was given one point, while there was no penalty or negative marking for the incorrect response.

Item analysis

Stem-type multiple-choice questions with a single best choice of four options were defined as an item in this study. The item difficulty index refers to the percentage of the total number of correct responses to the test item, calculated by the following formula [7-12]:

$Difficulty Index (P score) = \frac{total true response of the item}{total responses}\times 100$

where $total responses= (true response + wrong response + not responded)$

Item Discrimination Index

The total score of each examinee was calculated and arranged in descending order from highest score to lowest score. The upper one-fourth of the students (highest scorer of 100 students out of 400) were selected to include in the higher group (H), and the lower one-fourth of the students (lowest scorer of 100 students out of 400) were selected to include in the lower group (L). The item discrimination index was calculated by the following formula [7-12]:

$Item Discriminating Index (D Score) =\frac{HT-LT}{T}\times 2$

Where HT = number of correct responses in the upper group, LT = number of correct responses in the lower group, and T= total number of responses in both groups.

Distractor Discriminating Power

 Discriminating power for each distractor was calculated by the following formula [7-12]:

$Discriminating Power for Distractor = \frac{HR-LR}{T}\times 2$

Where HR = number of responses from the upper group, LR = number of responses from the lower group, and T= total number of responses

Distractor effectiveness for the option indicates the percentage of students choosing the option of the item as an answer. Distractor effectiveness ranges from 0 to 100%, which depends on the number of functioning distractors. MCQs in this study had four options, so distractor effectiveness for items with one, two, and three functioning distractors was 33.33%, 66.66%, and 100%, respectively.

Good distractors appeal to a higher proportion of low-achieving examinees when compared with high-achieving examinees, thereby resulting in a negative statistic [14]. A non-functioning distractor was defined as an option with either a response frequency of <5% or positive discriminating power. In this study, each item had four options, so the maximum number of functioning distractors for each item was three and the minimum number of functioning distractors for each item was zero.

Data analysis

Calculated item difficulty index, item discriminating index, distractor discriminating power, and the number of functioning distractors for each item were compiled in a data sheet with the help of Microsoft Office Excel 2017.

The data were analyzed statistically for the relationship between the number of functioning distractors, the item difficulty index, and the item discriminating index using Epi Info 7 TM Software.

## Results

The test paper, with 200 items responded to by 400 examinees, was analyzed for objectives. The KR20 reliability index of the test paper was 0.91 (>0.70), and the data distribution curve was normal. The mean item difficulty index was 57.7575% ± 28.6594% (95% confidence interval (CI), p<0.05); the minimum item difficulty index was 26.5% and the maximum was 89%.

The mean item discriminating power was 0.4388 ± 0.3664 (95% confidence interval, p<0.05), while the minimum item discriminating power was 0.04 and the maximum was 0.83.

Five hundred and five out of 600 distractors were functioning (distractor effectiveness=84.17%), while not a single item had 0% distractor effectiveness. The response frequency of 46 (7.67%) distractors was less than 5.00%, while 66 (11%) distractors had positive distractor discrimination power (responded more by high-achieving examinees than low-achieving examinees).

One hundred and twenty-three out of the total of 200 items (61.50%) had three functioning distractors, while 59 (29.50%) items had two functioning distractors, and 18 (9.00%) items had only one functioning distractor (Table 1).

**Table 1:** Item difficulty index and item discriminating power according to the functioning distractors per item

Number of functional distractors	Frequency	Mean item difficulty index	Mean item discriminating index
1	18 (9.00%)	74.9167%	0.2283
2	59 (29.50%)	56.3305%	0.3558
3	123 (61.50%)	55.9309%	0.5093
Total	200 (100.00%)	57.7575%	0.4388
Confidence interval = 95%, P score< 0.05			

**Figure 1:**
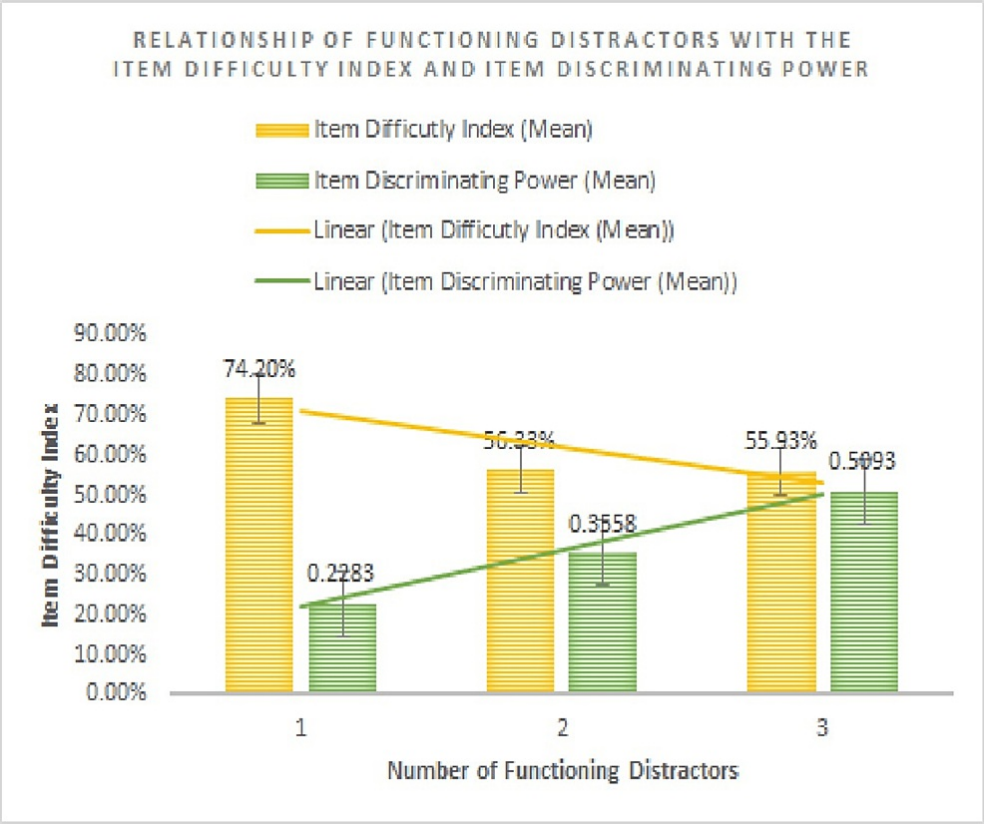
Relationship of the number of functioning distractors with item difficulty index and item discriminating power

The items were divided into three groups on the basis of the number of functioning distractors (Table 1, Figure 1): items with one, two, and three functioning distractors. Analysis of variance (ANOVA) shows a significant difference in the item difficulty index among the items with one, two, and three functioning distractors (F value=440.1308, p-value=0.004, confidence interval=95%, Figure 1).

The item difficulty index was found to be higher in items with a single functioning distractor in comparison to items with three functioning distractors (Tukey (honest significant difference) HSD post hoc test, diff=-18.9858, 95% CI=-20.5194 to -17.4522, p<0.005) and with two functioning distractors (Tukey HSD post hoc test, diff=-18.9858, 95% CI=-20.5194 to -17.4522, p=0.0000). There was no significant difference in the item difficulty index between items with two functioning distractors and items with three functioning distractors (Tukey HSD post hoc test, diff=-0.3996, 95% CI=-1.3620 to 0.5628, p=0.5901).

The ANOVA test shows a significant difference in the mean item discrimination index among the items with one, two, and three functioning distractors (F value=4.912, p-value=0.008, confidence interval=95%).

The item discriminating index was found to be higher in items with a single functioning distractor in comparison to items with three functioning distractors (Tukey HSD post hoc test, diff=0.2810, 95% CI=0.0249 to 0.5371, p=0.0277). There was no significant difference in the item discriminating index between items with two functioning distractors and items with three functioning distractors (Tukey HSD post hoc test, diff=0.1535, 95% CI=-0.0072 to 0.3142, p=0.0647), or between items with one and two functioning distractors (Tukey HSD post hoc test, diff=0.2810, 95% CI=0.0249 to 0.5371, p=0.0277).

## Discussion

In this study, the mean item difficulty index, mean item discriminating power and distractor effectiveness were 57.7575%, 0.4388, and 84.17%, respectively, which is in the ideal range and correlates with other studies [15-18].

MCQs with four options are commonly used for competitive examinations and formative assessment, so this study used a test paper consisting of MCQs with four options. Raahma et al. (2017) reported in their study that MCQs with four options are more reliable than those with three options [19]. Fozzard et al. (2018) did not find a significant difference in the item difficulty index and item discriminating power among MCQs with four options and five options [20]. London C. et al. (2018) reported that MCQs with four or five options are good discriminators and serve the role of exams [21]. Constructing a MCQ with four functioning options is challenging even for an experienced examiner; increasing options to more than four increases the chances of having a nonfunctioning distractor [21-23]. Increasing the number of options to more than four reduces the probability of selection of a distractor by an examinee by less than 25%; in addition, some distractors become obviously ruled out, which makes the distractor non-functional. MCQs with only two or three options increase the chances of a correct answer by using guessing methods.

This study shows a negative linear relationship between the item difficulty index and the item discriminating power, which correlates with previous studies [17,22,23]. This result indicates tough items are good discriminators while easier items are poor discriminators; it may be due to lower achievers not being able to correctly answer the tough items while easier items can be answered correctly by both high and low achievers.

Not a single item in this study had 0.00% distractor effectiveness, and only 18 items had 33.33% distractor effectiveness, which indicates all MCQs were constructed with appropriate distractors. The item difficulty index was higher for items with only one functioning distractor in comparison to items with all three functioning distractors. Similarly, items with all three functioning distractors had higher discriminating power than items with only one functioning distractor. Items with all three functioning distractors were successful in assessing the knowledge difference between high and low achievers; that indicates constructing proper distractors fulfills the objective of the examination.

Although the item difficulty index does not show a significant difference between items with two functioning distractors and items with all three functioning distractors, the item discriminating power differs among items with two functioning distractors and items with all three functioning distractors. Constructing every distractor is important to differentiate high achievers from low achievers; even if two distractors are constructed properly, it may maintain the ideal difficulty index.

MCQ is constructed to assess the knowledge of the examinee on a specific topic, while a group of MCQs or a test paper needs to differentiate knowledge among a group of examinees. Setting a test paper requires including MCQs with low, acceptable, and high difficulty indexes, but all MCQs in the test papers should have item discriminating power acceptable to the ideal range. For improving the item's discriminating power, more attention should be given to the construction of distractors in addition to stem formations.

## Conclusions

Item discriminating power is affected by every non-functioning distractor, which makes it very important to discriminate between high-achieving and low-achieving examinees. Two or three functioning distractors show an appropriate item difficulty index and item discriminating power. In addition, item discriminating power is lower for the easy MCQs. To improve and maintain the quality of MCQ, non-functioning distractors should be identified and removed or replaced with functioning distractors to maintain the number of functioning distractors between two and three.

## References

[1] Hubbard JP, Clemans WV (1961). Multiple-choice Examinations in Medicine. A Guide for Examiner and Examinee. Journal of Medical Education.

[2] Skakun EN, Nanson EM, Kling S, Taylor WC (1979). A preliminary investigation of three types of multiple choice questions. Med Educ.

[3] Kehoe J (1994). Basic item analysis for multiple-choice tests. Practical Assessment, research, Evaluation.

[4] De Champlain AF, Melnick D, Scoles P (2003). Assessing medical students' clinical sciences knowledge in France: a collaboration between the NBME and a consortium of French medical schools. Acad Med.

[5] National Testing Agency (12 March, 2021 2021 (2023). National Eligibility Cum Entrance Test. UG.

[6] Zainudin S, Ahmad K, Ali NM, Zainal NF (2012). Determining course outcomes achievement through examination difficulty index measurement. Procedia - Social and Behavioral Sciences.

[7] Zubairi AM, Kassim NLA (2006). Classical and Rasch analysis of dichotomously scored reading comprehension test items. Malaysian Journal of ELT Research.

[8] Considine J, Botti M, Thomas S (2005). Design, format, validity and reliability of multiple choice questions for use in nursing research and education. Collegian.

[9] Miller MD, Linn RL, Gronlund NE, editors editors (2009). Measurement and Assessment in Teaching. https://www.worldcat.org/title/measurement-and-assessment-in-teaching/oclc/174501624.

[10] Fowell SL, Southgate LJ, Bligh JG (1999). Evaluating assessment: the missing link?. Med Educ.

[11] Tarrant M, Ware J, Mohammed AM (2009). An assessment of functioning and non-functioning distractors in multiple-choice questions: a descriptive analysis. BMC Med Educ.

[12] Dufresne RJ, Leonard WJ, Gerace WJ (2002). Making sense of student’s answers to multiple-choice questions. Phys Teach.

[13] Haladyna TM, Downing SM (1989). Validity of taxonomy of multiple-choice item-writing rules. Appl Meas Educ.

[14] McCoubrie P (2004). Improving the fairness of multiple-choice questions: a literature review. Med Teach.

[15] Bhat SK, Prasad KH (2021). Item analysis and optimizing multiple-choice questions for a viable question bank in ophthalmology: a cross-sectional study. Indian J Ophthalmol.

[16] Raymond MR, Stevens C, Bucak SD (2019). The optimal number of options for multiple-choice questions on high-stakes tests: application of a revised index for detecting nonfunctional distractors. Adv Health Sci Educ Theory Pract.

[17] Gajjar S, Sharma R, Kumar P, Rana M (2014). Item and test analysis to identify quality multiple choice questions (MCQs) from an assessment of medical students of Ahmedabad, Gujarat. Indian J Community Med.

[18] Chauhan PR, Chauhan GR, Chauhan BR, Vaza JV, Rathod SP (2015). Relationship between difficulty index and distracter effectiveness in single best-answer stem type multiple choice questions. Int J Anat Res.

[19] Rahma NA, Shamad MM, Idris ME, Elfaki OA, Elfakey WE, Salih KM (2017). Comparison in the quality of distractors in three and four options type of multiple choice questions. Adv Med Educ Pract.

[20] Fozzard N, Pearson A, du Toit E, Naug H, Wen W, Peak IR (2018). Analysis of McQ and distractor use in a large first year health Faculty Foundation program: assessing the effects of changing from five to four options. BMC Med Educ.

[21] Loudon C, Macias-Muñoz A (2018). Item statistics derived from three-option versions of multiple-choice questions are usually as robust as four- or five-option versions: implications for exam design. Adv Physiol Educ.

[22] Hingorjo MR, Jaleel F (2012). Analysis of one-best MCQs: the difficulty index, discrimination index and distractor efficiency. J Pak Med Assoc.

[23] Chauhan PR, Rathod SP, Chauhan BR, Chauhan GR, Adhvary A, Chauhan AP (2013). Study of difficulty level and discriminating index of stem type multiple choice questions of anatomy in Rajkot. Biomirror.

